# Development and Validation of a Non-Invasive, Chairside Oral Cavity Cancer Risk Assessment Prototype Using Machine Learning Approach

**DOI:** 10.3390/jpm12040614

**Published:** 2022-04-11

**Authors:** Neel Shimpi, Ingrid Glurich, Reihaneh Rostami, Harshad Hegde, Brent Olson, Amit Acharya

**Affiliations:** 1 Marshfield Clinic Research Institute, Marshfield, WI 54449, USA; shimpi.neel@marshfieldresearch.org (N.S.); glurich.ingrid@marshfieldresearch.org (I.G.); 2 Computer Science Department, University of Wisconsin-Milwaukee, Milwaukee, WI 53211, USA; reyhane.rostami@gmail.com; 3 Lawrence Berkeley National Laboratory, Berkeley, CA 94720, USA; hegdehb@gmail.com; 4 Office of Research Analytics and Computing, Marshfield Clinic Research Institute, Marshfield, WI 54449, USA; olson.brent@marshfieldresearch.org; 5 Advocate Aurora Health, Chicago, IL 60515, USA

**Keywords:** oral cancer, precision medicine, machine learning, risk assessment, patient care management

## Abstract

Oral cavity cancer (OCC) is associated with high morbidity and mortality rates when diagnosed at late stages. Early detection of increased risk provides an opportunity for implementing prevention strategies surrounding modifiable risk factors and screening to promote early detection and intervention. Historical evidence identified a gap in the training of primary care providers (PCPs) surrounding the examination of the oral cavity. The absence of clinically applicable analytical tools to identify patients with high-risk OCC phenotypes at point-of-care (POC) causes missed opportunities for implementing patient-specific interventional strategies. This study developed an OCC risk assessment tool prototype by applying machine learning (ML) approaches to a rich retrospectively collected data set abstracted from a clinical enterprise data warehouse. We compared the performance of six ML classifiers by applying the 10-fold cross-validation approach. Accuracy, recall, precision, specificity, area under the receiver operating characteristic curve, and recall–precision curves for the derived voting algorithm were: 78%, 64%, 88%, 92%, 0.83, and 0.81, respectively. The performance of two classifiers, multilayer perceptron and AdaBoost, closely mirrored the voting algorithm. Integration of the OCC risk assessment tool developed by clinical informatics application into an electronic health record as a clinical decision support tool can assist PCPs in targeting at-risk patients for personalized interventional care.

## 1. Introduction

Oral cancers represent the largest subtype of head and neck cancers. Oral cancers include cancers that arise in the oral cavity and pharynx [1]. American Cancer Society Surveillance research predicts that nearly 54,000 individuals will be newly diagnosed with oral cancers in the United States in 2022, including oral cavity cancer (OCC) and oropharyngeal cancer (OPC) [2]. Five-year survival rates were projected at approximately 60%, with an estimated 11,230 deaths attributable to oral cancer in the same year [2]. Global incidence estimates are similarly high, reflecting rates of greater than 377,713 cases of newly diagnosed patients annually [3].

Currently, oral cancer detection often occurs only at advanced stages. Late-stage diagnosis accounts for high morbidity and mortality. Mortality rates were estimated at 44% following 5-year survival rate analyses [4]. Notably, survival rates as high as 80% to 90% have been projected in the context of implementing reliable risk assessment and screening in the clinical setting to promote early-stage diagnosis [4]. Prevention, early detection, and intervention are projected to decrease mortality while simultaneously reducing morbidity associated with late-stage interventional treatment. Early detection and intervention are associated with improved outcomes, quality of life for patients, and reduction in associated medical and societal tolls [5].

Although tobacco and alcohol consumption and human papillomavirus are recognized as the most common risk factors of oral cancer [6], other candidate causal factors have been advanced, including lesions around the mouth, nodular leukoplakia, protracted irritations in the mouth (e.g., poorly fitted dentures), and poor diet, among others [7,8,9]. Molecular studies have estimated that malignant transformation associated with oral cancers involves three to six somatic mutations [10,11]. Studies have shown that approximately 72% of head and neck cancers involve a chromosomal deletion in the 9p21-22 region [8,12]. This deleted region is also noted in dysplastic and carcinoma in situ lesions, suggesting regional involvement during the early stages of carcinogenesis [12]. However, such genetic testing is not routinely performed for oral cancer patients; hence, determining patient risk for OCC currently relies mainly on monitoring environmental factors such as tobacco and alcohol exposure.

PCPs play a major role in engaging patients in health education, coordinating care, and referring patients to other specialties [13,14]. However, several studies have identified gaps in training provided to PCPs surrounding the performance of oral examinations and oral cancer assessments [13]. Historical evidence also reveals a low frequency of oral examinations by PCPs compared to examination rates by dental providers [14,15]. Moreover, complex relationships and convergent interactions among multiple risk factors for oral cancer make risk assessment at point-of-care (POC) difficult for PCPs. Leveraging the application of effective data mining approaches [16,17] to formidable amounts of historical clinical and demographic data captured during the patient visit in medical and dental records will allow the development of clinical decision support tools (CDSTs) trained in the identification of phenotypic characteristics associated with patients at highest risk for oral cancer. When embedded in medical records, CDSTs are powerful support tools for flagging patients to providers at risk for oral cancer and potentially in need of further assessment and intervention [18].

The application of machine learning (ML) to large data sets for the purpose of generating algorithms has proven highly successful in defining risk factors that can be monitored and systematically analyzed electronically to project attributable risk for disease emergence [19]. ML has been applied previously for the purposes of predicting and diagnosing various types of cancers or estimating the cancer survivability in the context of bladder cancer [20,21], breast cancer [22,23], colon cancer [24,25], and lung cancer [26,27]. Established frameworks for cancer detection and prevention have been proposed for the development of intelligent systems applying data mining techniques and ML algorithms [28,29]. The application of ML algorithms has been previously leveraged to identify causal factors of oral cancer, predict oral cancer survivability, and estimate the future risk of oral cancers [30,31]. The current study sought to extend methodological approaches to achieve and implement the most appropriate oral cancer risk assessment tool with the highest applicability to our clinic population to support the creation of the CDST prototype. The use of rich data within our integrated medical–dental electronic health record (iEHR) was proposed for the development of the oral cancer CDST [32]. The envisioned tool functionality included the capacity to evaluate relative risk, achieve early detection to promote improved survival rates among patients impacted by oral cancer, and support prevention. The approach to the development and validation of our risk assessment tool prototype and relative performance compared to previously developed tools is discussed.

## 2. Materials and Methods

Marshfield Clinic Health System (MCHS) ranks among the largest private practice groups in the country, providing multispecialty care through a network of over 50 regional medical clinics and 10 dental clinics across an expansive, largely rural service area spanning central, northern, and western Wisconsin. The very stable population residing within the MCHS service area is >95% white and largely of northern European descent. Racial and ethnic inclusion of patients within the dataset analyzed in the current study was limited to white, non-Hispanic individuals, since other races and ethnicities are underrepresented in the MCHS service area.

Historical data spanning a 35-year temporal period captured in the MCHS data warehouse were collected, preprocessed, and prepared. The prepared datasets were subsequently used to train potential classifiers. The classifier exhibiting the highest performance measures was selected to be at the core of the risk assessment tool. Novel data (validation set) not used to develop the classifier were then presented to the oral cancer risk assessment tool (OCRAT) to validate its performance. The predicted class calculated and reported by the tool for these novel data were classified as ‘cancer’ or ‘non-cancer’, reflecting the relative risk of the patient developing oral cancer in the future based on patterns of reported environmental exposures and other clinical factors captured in the iEHR. Java programming language and Weka library were used to develop OCRAT [33,34].

### 2.1. Data Preparation

Incident oral cancer cases diagnosed between a temporal 35-year window were identified in MCHS’ enterprise data warehouse (EDW). Patients with no prior history of an oral cancer diagnosis in this temporal window were age-and gender-matched to cases in a one-to-one ratio. Cases with salivary gland tumors and pharynx tumors were considered ineligible to restrict inclusion to only oral cavity cancer (OCC). Out of an eligible pool of >2300 patients with oral cancer (cases), a subset of 526 cases and an equal number of 526 controls (patients with no diagnosis of oral cancer) were selected. Enrollment was limited to non-Hispanic/Latino ethnicity and white/Caucasian race. Following an extensive literature review, a comprehensive list of variables representing potential candidates associated with OCC was specified for collection and analysis in the dataset. Data deletion was carried out where the percentage of missing data for each variable and percentage of missing data for each record were calculated. After data deletion, the following features were retained: tobacco use, alcohol abuse, disease of lips, esophageal reflux, oral aphthae, disorders of oral soft tissue, stomatitis and mucositis, oral leukoplakia, swelling or lump in the mouth, radiation therapy exposure or chemotherapy exposure for any cancer type, throat pain, and oral thrush.

### 2.2. Feature Ranking

A feature-ranking algorithm was applied to order the risk factors based on their relative contribution to risk for OCC emergence and generate various new data models using a dimensionality reduction technique. “Gain ratio” was applied as a measure of effectiveness in classifying and ranking the dataset into two classes. This statistical property is obtained through measuring entropy, an indicator of purity/impurity of data, and information gain that determines how significant a feature is in providing a good classification. Entropy of collection S is calculated as follows:

(1) $$Entropy\left(S\right)\equiv \overset{c}{\underset{i=1}{\sum }}-p_{i}\log_{2}p_{i}$$

where $p_{i}$ represents the proportion of examples in S. that belong to class i, and c is the number of classes. The information gain of attribute *A* of *S*, *Gain* (*S*,*A*), is defined as:

(2) $$Gain\left(S,A\right)\equiv Entropy\left(S\right)-\underset{v\in Values\left(A\right)}{\sum }\frac{\left|S_{v}\right|}{\left|S\right|}\;Entropy\left(S_{v}\right)$$

where values (*A*) represent all the values that attribute (*A*) can take, and $\;S_{v}=\left\{s\in S|A\left(s\right)=v\right\}$. The gain ratio considers information gain, as well as information regarding the branches of the decision tree [34]. A higher gain ratio corresponds to the higher accuracy of the classifier.

### 2.3. Dimensionality Reduction

In this step, dimensionality reduction was applied to generate 12 data models from our dataset. Dimensionality reduction refers to a process that reduces the number of features in a dataset using techniques such as feature selection (Sharma and Om, 2015). This method selects candidate features based on a specified criterion. Specifically, the gain ratio is the target feature in this instance. Rather than analyzing only the original dataset with 1052 cases and controls, we generated 12 data models from the dataset and presented all of them to our data analysis pipeline. The first data model contained all 13 OCC risk factors. To generate the second data model, the attribute with the lowest gain ratio was eliminated to arrive at a model limited to the 12 top-ranking risk factors exhibiting the highest gain ratios. The third data model again eliminated the attribute with the lowest gain to arrive at 11 features. By eliminating features and generating the data models with n-1 attributes, ultimately, only two features remained in the twelfth data model. The rationale supporting model generation by this process was to establish the relative contribution of different risk factors to OCC emergence and define the most highly accurate prediction model informed by the minimum number of the most predictive features. This approach enabled us to evaluate risk even in the context of a limited number of highly predictive variables.

### 2.4. Data Analysis

Six ML algorithms were used as classifiers to implement OCRAT. The classifiers were multilayer perceptron (MLP), k-nearest neighbor (KNN), decision tree (DT), AdaBoost, radial basis function (RBF) networks, and voting algorithm (which uses DT, RBF, and MLP classifiers). The 12 data models were used to train the classifiers. The performance of classifiers was evaluated and compared using four metrics: accuracy, precision, specificity, and sensitivity (recall), as well as by plotting receiver operating characteristic (ROC) and recall–precision (RP) curves.

These metrics were calculated through the creation of a confusion matrix and measurement of true-positive (TP), true-negative (TN), false-positive (FP), and false-negative (FN) values. For each classifier, the confusion matrix was formed, and then four metrics were calculated as follows:

Accuracy = TN + TP/(TP + TN + FP + FN)(3)

Recall (Sensitivity) = TP/(TP + FN)(4)

Precision = TP/(TP + FP)(5)

Specificity = TN/(TN + FP)(6)

The performance of classifiers was reported based on a 10-fold cross-validation technique used to train and test the classifiers. The criterion for selecting the ideal data model was informed by identifying the algorithm with the highest accuracy and recall for the classifiers used in the data model. Precision and specificity metrics were evaluated as further comparisons. This step resulted in the elimination of some classifiers in subsequent analyses based on the outcome following the calculation of the areas under the ROC (AUC) and RP curves. The best predictive model for the tool was determined by selecting the model with the highest area under both the AUC and RP curves. The Weka software package (version 3.6.12) was used to implement the classifiers [34].

### 2.5. Sensitivity Analysis

Once defined, the classifier with the highest performance was evaluated for reliability by conducting a sensitivity analysis designed to define fluctuations in the output of the model with respect to the variability in the input [35]. The sensitivity analysis was performed applying the mathematical method described by Yao (2003) [36]. To this end, besides the original training dataset (526 cases and 526 controls), 10 additional validation datasets equivalent in size to the control data set (*n* = 526) were randomly selected from the initial control dataset. These control datasets, along with the 526 cases, formed 10 new datasets, effectively achieving a sample size of 1052 subjects that was used for the purpose of conducting sensitivity analysis. Twelve data models were again generated for each of these additional 10 datasets. The entire data analysis process was applied to both the original and 10 additional datasets.

## 3. Results

The ranking of the features based on their gain in the original dataset of 526 cases and controls is presented in Table 1.

**Figure 1:**
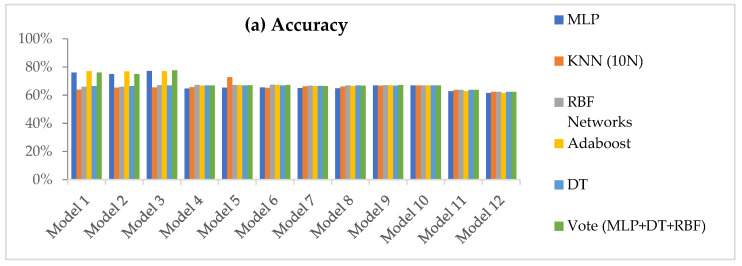

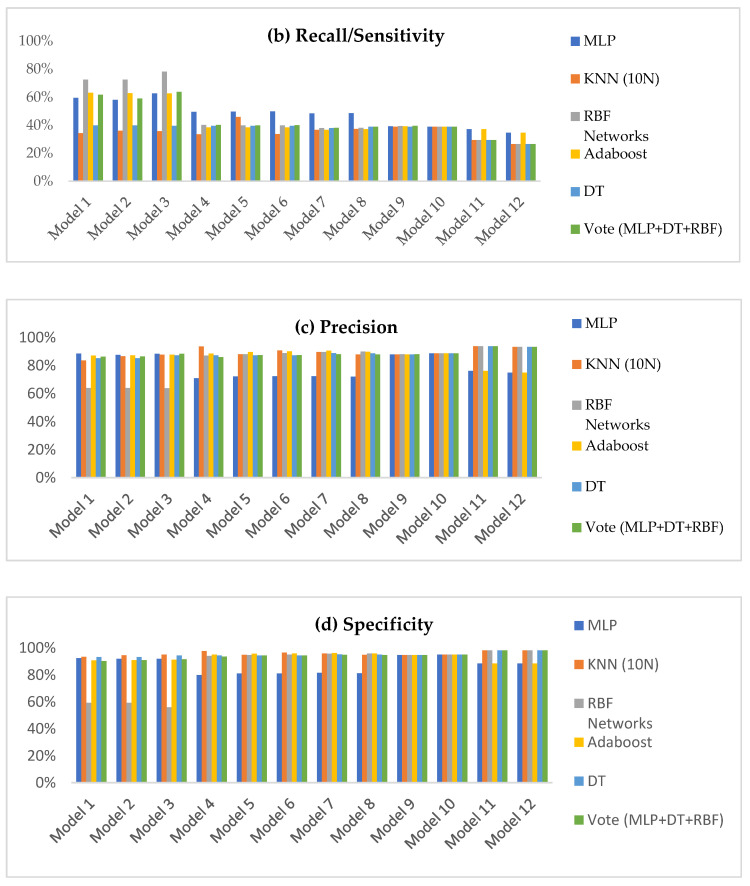
Summary of the performance metrics for six classifiers: (**a**) Accuracy; (**b**) Recall/Sensitivity; (**c**) Precision and (**d**) Specificity.

In total, 12 data models were generated for 11 datasets ordered by their ranking, and the 6 candidate classifiers were applied to all of them. Figure 1 summarizes 792 comparisons (11 datasets × 12 data models × 6 classifiers), with comparisons across the classifiers representing their accuracy, recall (sensitivity), specificity, and precision.

**Figure 2:**
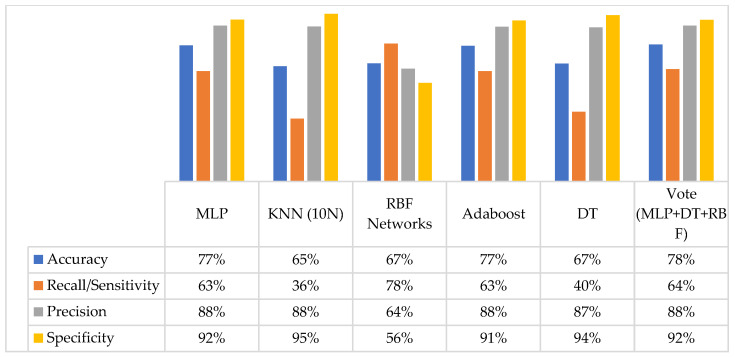
Performance of the classifiers in terms of four evaluation metrics on third model.

Model 3 represents the highest accuracies for all classifiers compared with the other 11 models. The accuracies for MLP, KNN, RBF networks, AdaBoost, DT, and voting algorithm are 77%, 65%, 67%, 77%, 67%, and 78%, respectively. In Figure 1b, Model 3 achieved the highest recall rate for all classifiers except MLP and AdaBoost. Figure 2 shows the performance of the classifiers for the four evaluation metrics of the third model.

**Figure 3:**
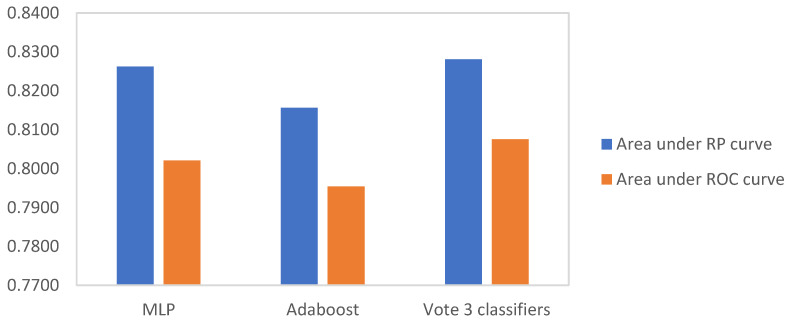
Area under RP curve and ROC curve for MLP, AdaBoost, and voting algorithm.

Due to poor recall, KNN (36%) and DT (40%) were not deemed appropriate candidates to support OCRAT. RBF networks showed the highest recall value. However, due to its low specificity, it was eliminated from the next round of measurements. The remaining classifiers, MLP, AdaBoost, and voting algorithm, were investigated in more detail by applying ROC and RP curves. The calculated AUC for the ROC and RP curves for MLP, AdaBoost, and the voting algorithm are illustrated in Figure 3.

**Figure 4:**
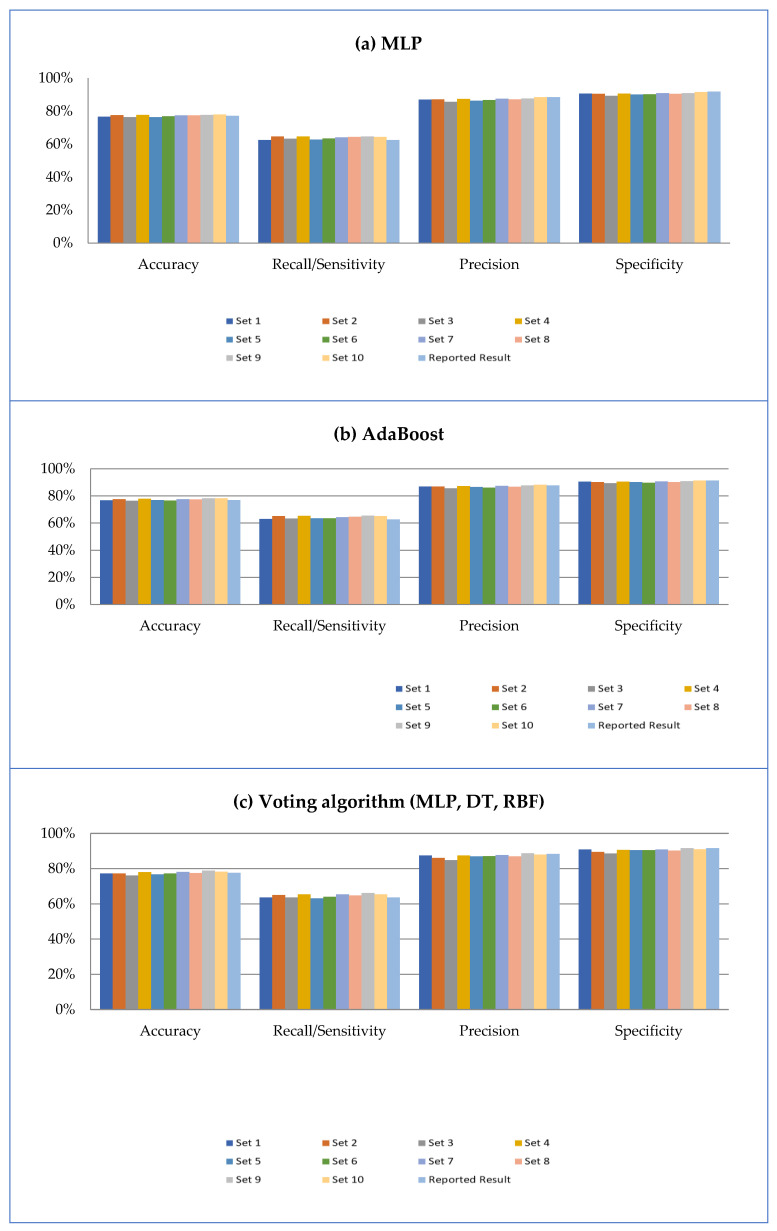
Result of applying (**a**) MLP, (**b**) AdaBoost, and (**c**) voting algorithm on third model.

The voting algorithm emerged as the superior classifier in comparison to MLP and AdaBoost, exhibiting the greatest AUC while achieving 78% accuracy, 64% recall, 88% precision, and 92% specificity. The results of the sensitivity analysis are summarized in Figure 4.

As observed in Figure 4, MLP and AdaBoost performance closely mirrored that of the voting algorithm. The fluctuations between accuracy, sensitivity, specificity, and precision analyses for three classifiers were <3%, as shown in Figure 4a–c.

## 4. Discussion

We developed OCRAT as a CDST prototype using ML algorithms with the capacity to profile variables associated with high-risk oral cancer phenotypes using ML algorithms with the capacity to profile variables associated with high-risk oral phenotypes. Frequency matching and sensitivity analysis methods that ensure the reliability of the experimental results were used in our proposed system. Several classifiers, namely MLP, DT, KNN, AdaBoost, RBF networks, and voting algorithm, were explored. Three of these classifiers, KNN, AdaBoost, and voting, have not been applied to oral cancer risk assessment previously.

Notably, in the present study, low false-negative rates were exhibited by our model. In the context of risk prediction in the clinical setting, achievement of low false-negative rates is relatively more important than achieving low false-positive rates, because a low false-negative rate falsely attributes low risk to a patient actually at high risk. Failure to detect patients at high risk is associated with higher future costs due to ensuing morbidity and mortality associated with cancer emergence.

Machine learning has previously been applied to oral cancer risk assessment. Table 2 shows the comparisons of the performance metrics in terms of sensitivity and specificity across studies that applied ML methods for predicting OCC risk.

One type of ML algorithm termed ‘genetic programming’ was used to diagnose oral cancer [37,40]. Genetic programming methodology is based on the application of algorithms to evolutionary data that use the Darwinian principle of natural selection to address their goal. The data surrounding patients’ lifestyles collected from the questionnaires were used to train and test the proposed system. The prepared dataset created by Kent et al. [37] included 29 features. The study reported accurate diagnosis rates of 64% and 65% for cases and controls, respectively. Analysis of the classifier is shown in Table 2.

Tseng et al. [38] applied data mining techniques for oral cancer prognosis. Decision tree and ANN algorithms were used to analyze historical cases (673 cases, 426 controls specifying 23 risk factors) of oral cancer and predict the survival/mortality rates. The performance of classifiers was also compared with logistic regression and demonstrated that decision tree and ANNs outperformed the traditional approach [Table 2].

An early application of ML to oral cancer prediction reported the application of an artificial neural network (ANN) as a classifier to evaluate the likelihood of an individual having a malignant oral lesion based on the individual’s habits [31]. Rosma and colleagues [39] applied fuzzy NNs and fuzzy regression models to predict oral cancer risk. Their unmatched dataset included 84 cases and 87 controls. The accuracy of their model was 60%, and sensitivity and specificity are shown in Table 2.

By way of comparison, our work bears methodological similarity to the studies by Kent [37], Speight et al. [31], and Rosma et al. [39]. However, while Kent’s study [37] explored the use of genetic programming to diagnose oral cancer, our goal was to assess the future risk of developing OCC by defining variables contributing to high-risk phenotypes. Speight et al. [31] similarly engaged the goal of risk prediction using 29 risk factors that yielded approximately 65% accuracy, while our model exhibited improved accuracy of 78% using less than half the number of features. Speight et al. [31] included nine causal factors contributing to oral cancer in their analyses; six of them were related to smoking and alcohol consumption, each stratified across three levels of exposure (none, moderate, and heavy). Age, sex, and frequency of visiting a dentist were the three additional features. Their reported specificity was 77%, while our classifier achieved a specificity of 92%. However, the sensitivity of their classifier was 80%, 16% higher than that achieved by the voting algorithm in our work. This difference could be partially attributable to the richness of their dataset surrounding more complete data on smoking and alcohol exposure, two of the leading validated risk factors for OCC.

Voting techniques that use DT, RBF networks, and MLP classifiers outperformed the other classifiers in the current study and resulted in a robust assessment tool for predicting the future risk of developing oral cancer for new patients in the clinical setting. Further, the new classifiers tested in our work positively impacted risk assessment capacity. AdaBoost and voting algorithm, which have not been applied to oral cancer risk assessment previously, were among the superior classifiers. Overall, our third data model (Figure 1), which included the first 11 significant risk factors, outperformed other candidate models in our study, generating superior results while using only the 11 most significant features. Excellent performance was achieved despite the exclusion of alcohol abuse and chemotherapy. Moreover, the more abbreviated inventory of features has greater advantages in the clinical setting since the collection and evaluation of fewer self-reported data points during a new patient encounter adequately supports the CDST in attributing oral cancer risk across a range of highly predictive risk variables. Sensitivity analysis was also performed in this study to assess the relevance of the selected features. The difference between the highest and lowest matrix values of the three classifiers in Figure 4 was only 3%, indicating lack of bias toward a specific class and that outcomes observed were not due to random chance.

### Limitations

Some limitations, mainly attributable to the available data, are noteworthy. Lack of diversity in our dataset limited our results to the white race and non-Hispanic/Latino ethnicity. Our OCRAT tool may have low portability to these subpopulations where risk may vary with ethnicity and race. However, risk could not be meaningfully assessed in these subpopulations due to their underrepresentation.

Alcohol consumption represents an important risk factor contributing to oral cancer emergence [41]. However, in our study, incomplete data surrounding alcohol consumption, especially among the control group, proved to be a confounder to achieving higher sensitivity within our dataset. High rates of missing data, especially in control populations, impacted negatively on gain ratios and could not be improved by inferring data using tools in WEKA. Despite less-than-optimal data surrounding alcohol consumption and smoking, our tool nonetheless achieved acceptable sensitivity. While a study conducted by Rosma et al. [39] was also similar to ours, our results achieved substantially higher sensitivity compared to the relatively low sensitivity achieved by their fuzzy classifier.

Missing values surrounding important risk factors associated with oral cancer diminished the performance of the classifiers. Whereas tobacco consumption is a key established risk factor associated with OCC causation, deficits in the availability of structured data collected for tobacco consumption affected the classification results. Moreover, data surrounding other risk factors identified by the literature review, including premalignant conditions and lesions (such as thrush, recurrent mucositis, accretions on teeth, Vincent’s angina, stomatitis, oral aphthae, esophageal reflux), were missing or very limited. Due to the small number of cases for whom we have these data, we were not able to carry out further analyses for these variables. Oropharyngeal cancers are likely to be HPV related but are less commonly found in the context of OCC. In the present study, only found two cases were identified as having HPV high-risk status, and this number was insufficient for analytical inclusion. Hence, HPV positivity was not considered for further analyses. As genetic testing for OCC is not a routine practice, there is a lack of this information in the iEHR surrounding genetic alterations associated with OCC. Data on family history of OCC or head and neck cancer was also limited and was thus not included in the dataset.

## 5. Conclusions

Future plans to improve the tool’s predictive power will focus on exploring additional variables that can be gleaned from informative unstructured data. We plan to identify and extract additional new features from textual unstructured data found in the iEHR through natural language processing, adding it to our dataset and iteratively testing its relative contribution to oral cancer risk prediction [42].

## Figures and Tables

**Table 1:** Shows the features and their associated ranks.

Feature	Gain Ratio
Diseases of lips	0.1429
Disorders of oral soft tissue	0.1277
Leukoplakia in oral mucosa	0.1232
Presence of swelling or lump in mouth	0.0483
Throat pain	0.0398
Esophageal reflux	0.0287
Stomatitis and mucositis	0.0099
Radiation therapy	0.0087
Oral aphthae	0.0032
Oral thrush	0.0021
Tobacco use	0.0008
Chemotherapy	0.0003
Alcohol abuse	0.0000

**Table 2:** Performance metrics for various studies that used machine learning algorithms.

Reference No	N	ML Algorithms Used	Sensitivity	Specificity
Speight et al. [31]	1662	Neural network (NN)	80%	77%
Kent et al. [37]	1939	Genetic programming (GP) NN	73% GP 64% NN	65% GP 68% NN
Tseng et al. [38]	1099	Decision tree (DT) NN Logistic regression (LR)	Total accuracy: DT: 95.8% LR: 67.6% NN: 93.8%	
Rosma et al. [39]	191	Fuzzy NNs Fuzzy regression	46% fuzzy NN 69% fuzzy regression	85% fuzzy NN 65% fuzzy regression

## Data Availability

Not applicable.
